# Supplementary figures and images for: Systemic infection by Candida albicans requires FASN-α subunit induced cell wall remodeling to perturb immune response
Source: PLoS Pathog. 2025 Mar 26;21(3):e1012865. doi: 10.1371/journal.ppat.1012865 (PMC11940687; doi:10.1371/journal.ppat.1012865)

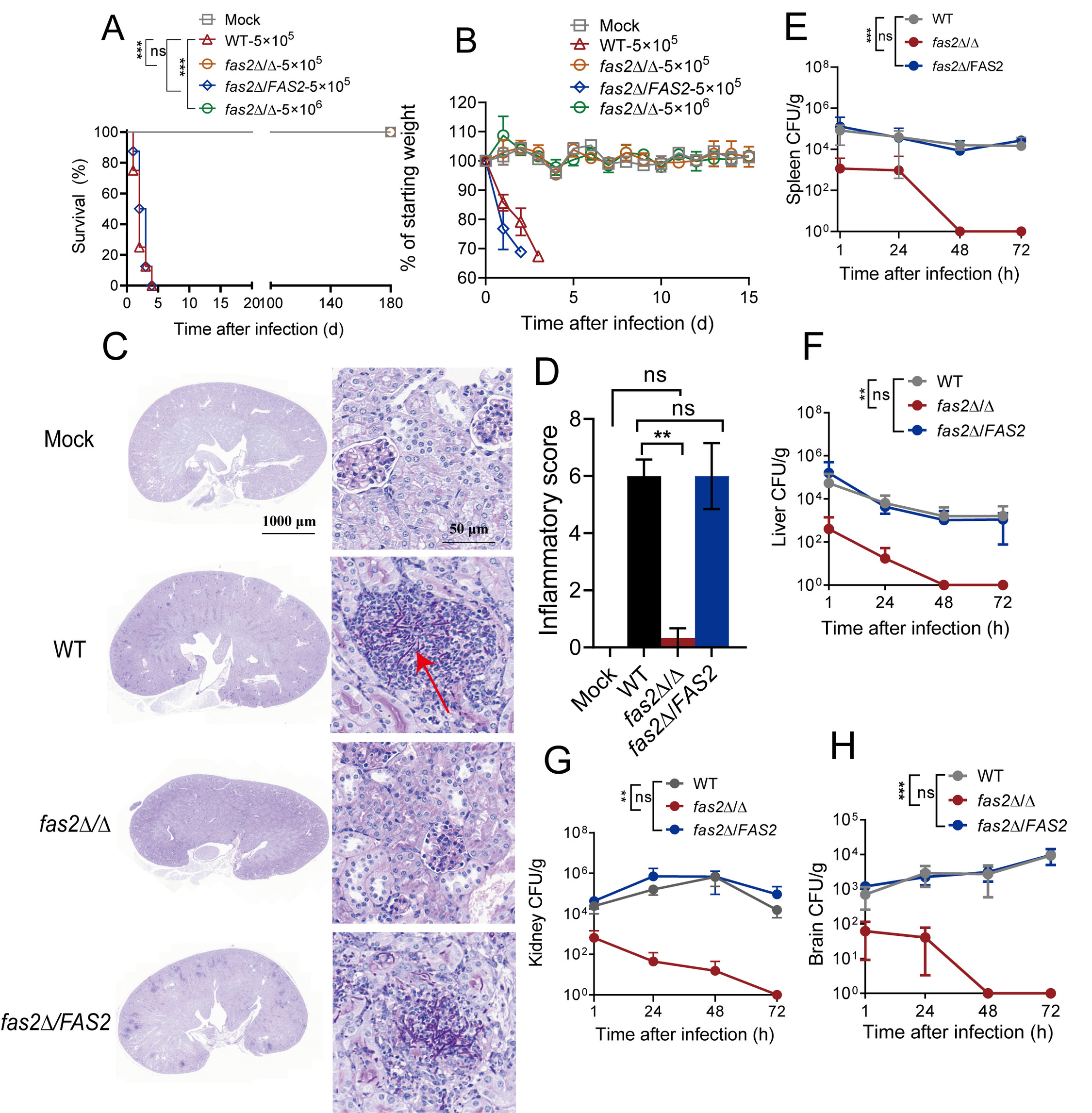

Supplement: S1 Fig — (A, B) Survival curves (A) and weight loss (B) of mice (n = 8) after intravenous injection with C. albicans WT, fas2Δ/Δ, fas2Δ/FAS2 at 5 × 10^5^ CFUs per mice, and fas2Δ/Δ at 5 × 10^6^ CFUs per mice. (C, D) Representative images of PAS stained kidney sections of mice (C) (n = 3) 72 h after systemic infection with WT, fas2Δ/Δ and fas2Δ/FAS2, and combined inflammatory score based on renal immune cell infiltration and tissue destruction (n = 3) after intravenous infection with C. albicans (D). (E-H) C. albicans fungal burdens (CFU per g of tissue) in organs at 1, 24, 48 and 72 h postinfection with WT, fas2Δ/Δ and fas2Δ/FAS2 (5 × 10^5^ CFUs per mice) (n = 3). Data in (A, B, D, E, F, and H) are presented as mean ± SD. Statistical significance was determined using the log-rank test for survival curves (A), two-tailed unpaired Student’s t-test for inflammatory score (D), and two-way ANOVA for fungal burdens (E, F, G, and H). Significance is indicated as *P < 0.05, **P < 0.01, ***P < 0.001, with ns denoting no significant difference. (TIF) [file ppat.1012865.s001.tif]

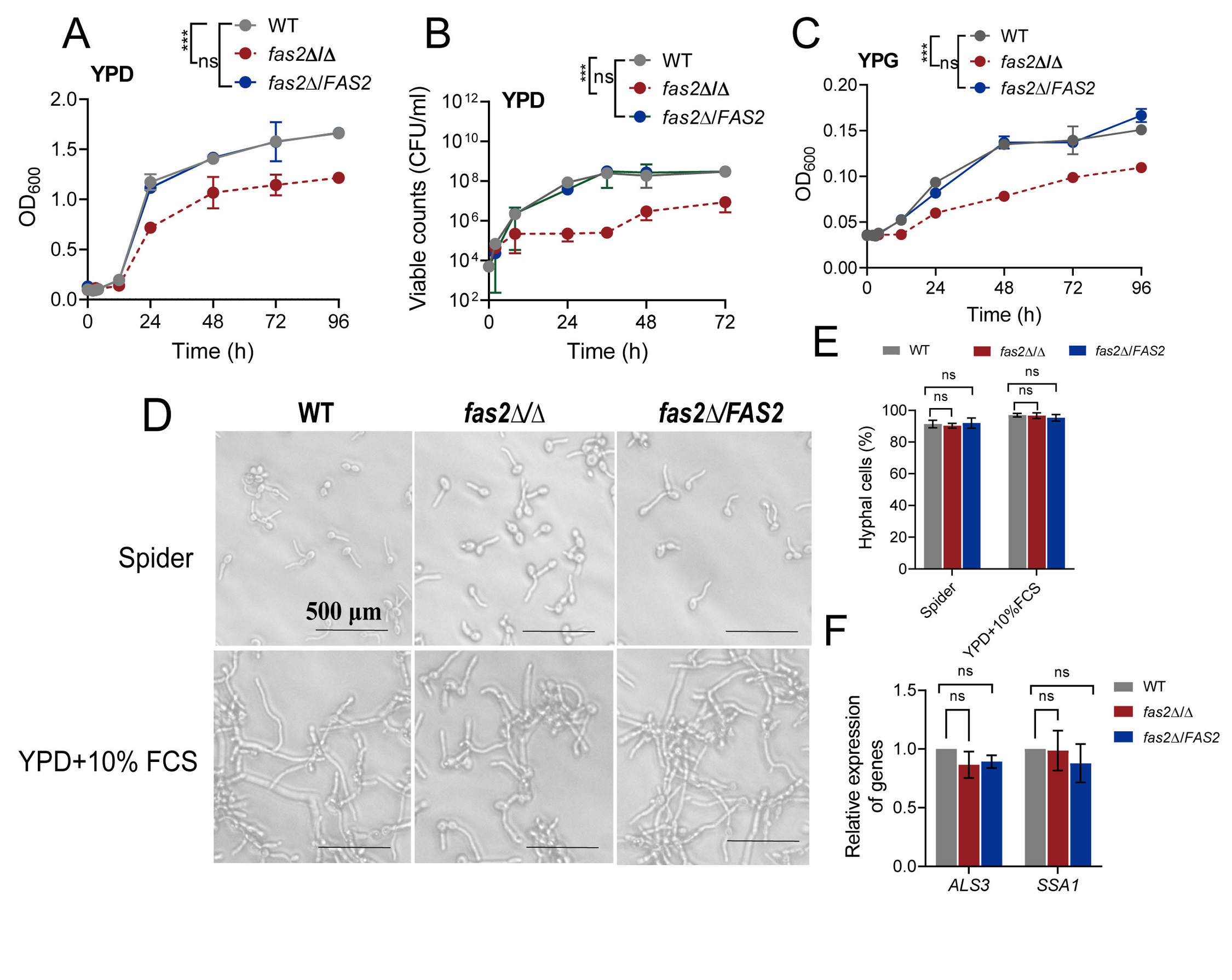

Supplement: S2 Fig — (A, B) The growth of WT, fas2Δ/Δ and fas2Δ/FAS2 in YPD (A, B), or YPS (C) media over time determined by cell density (OD600) (A, C) and viable cell counts (CFU/ml) (B). (D-F) Hyphae formation of WT, fas2Δ/Δ or fas2Δ/FAS2 strains was induced in Spider and 10% FCS liquid media for 2 h at 37 °C (D). In D magnification × 400. The percentage of hyphal cells (E) was calculated at least 100 cells in each group (n = 3). (F) The mRNA expression levels of the hyphae-associated genes of WT, fas2Δ/Δ or fas2Δ/FAS2 cultured in 10% FCS medium for 6 h as assessed by RT-qPCR. Data are expressed as the mean ± SD of three independent experiments. Statistical significance is indicated by *P < 0.05, **P < 0.01, ***P < 0.001, with ‘ns’ for not significant. Two-way ANOVA was used for statistical analysis in A-C; In D, one representative experiment out of three independent experiments is shown; the two-tailed unpaired Student’s t-test for E, and F. (TIF) [file ppat.1012865.s002.tif]

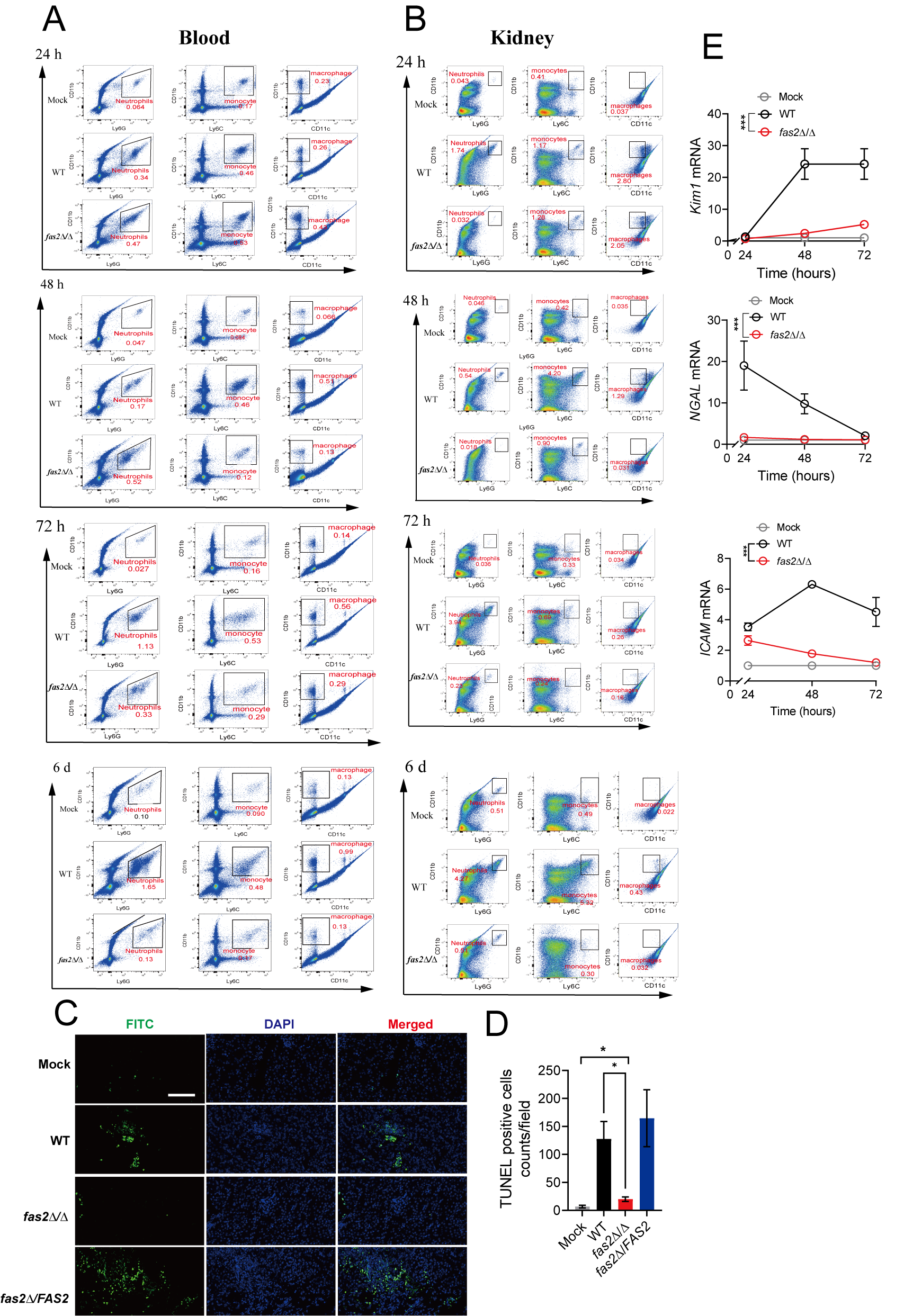

Supplement: S3 Fig — (A) Peripheral blood of mice was analyzed at 24, 48, 72 h, and 6 d after intravenous infection with C. albicans WT, fas2Δ/Δ, and fas2Δ/FAS2 (5 × 10^5^ CFUs per mice) using FACS, with cells stained with antibodies to CD11b, CD11c, Ly6c and Ly6G. One representative FACS plot per group (n = 3) was shown. (B) Kidney cells of mice were analyzed for immune cell populations at 24, 48, 72 h, and 6 d after intravenous infection with C. albicans WT, fas2Δ/Δ, and fas2Δ/FAS2 (5 × 10^5^ CFUs per mice) using FACS stained with antibodies to CD11b, CD11c, Ly6c and Ly6G (n = 3), and one representative experiment of three independent experiments is shown. (C, D) Representative images of TUNEL staining of mice infected with WT, fas2Δ/Δ and fas2Δ/FAS2 (C), and quantification of TUNEL-positive cells (D). (E) Kidneys were isolated for RT-qPCR analysis of indicated acute kidney injury genes or adhesion molecules expression after intravenous infection with C. albicans WT, fas2Δ/Δ, and fas2Δ/FAS2 (5 × 10^5^ CFUs per mice) (n = 3). Significance is indicated as *P < 0.05, **P < 0.01, ***P < 0.001, with ns denoting no significant difference. (TIF) [file ppat.1012865.s003.tif]

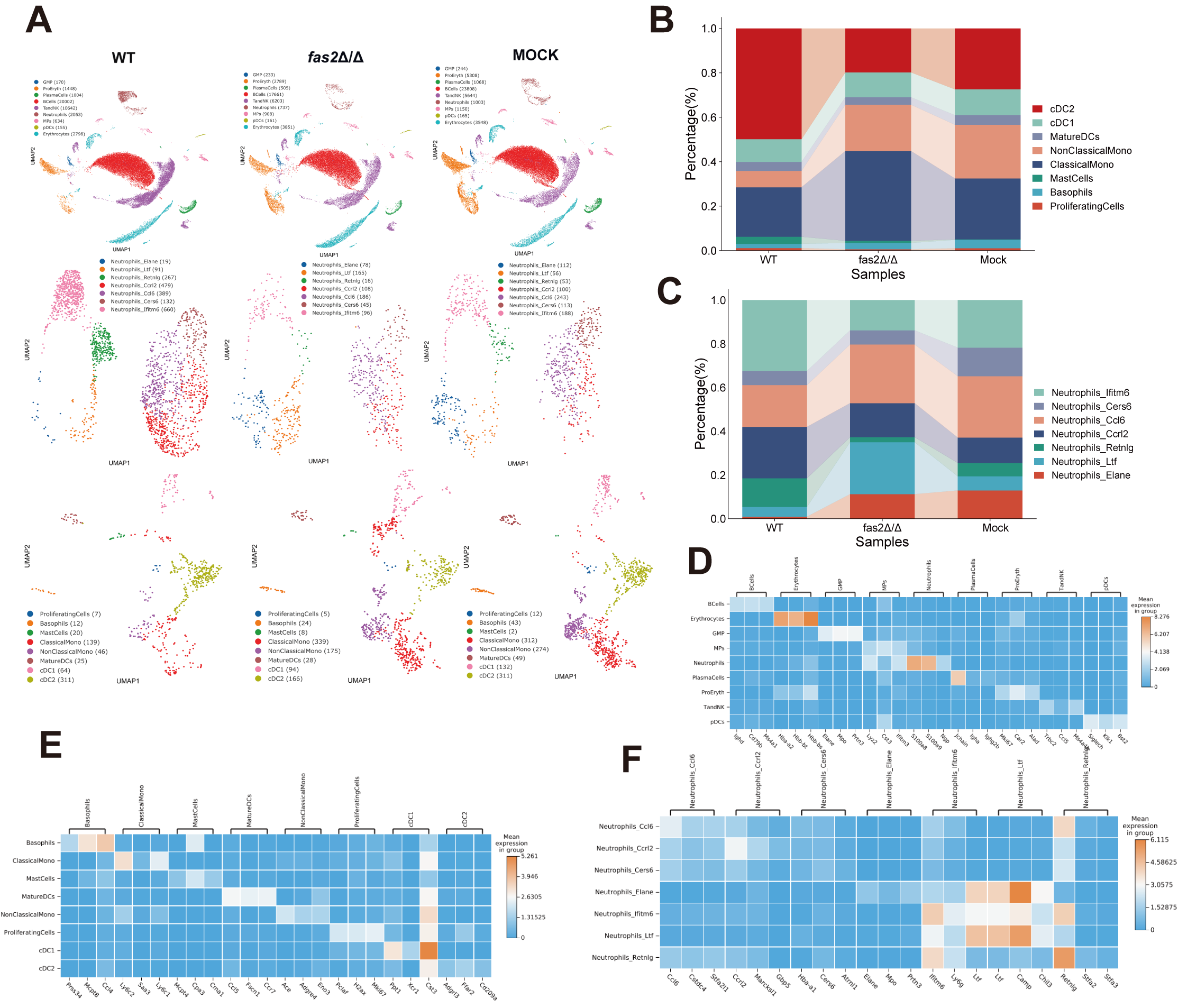

Supplement: S4 Fig — (A) UMAP plots depicting the clustering of splenic immune cells from WT, fas2Δ/Δ, and mock-infected mice. Each dot represents a single cell, colored according to its assigned cell type, with major populations including splenocytes, neutrophils, and monocyte phagocytes. Cells are partitioned into nine, seven, and eight distinct populations in WT, fas2Δ/Δ, and mock groups, respectively. (B) Stacked bar plot showing the relative abundance of monocyte phagocyte sub-clusters across WT, fas2Δ/Δ, and mock-infected samples (n = 3), with populations such as dendritic cells (cDC1, cDC2), macrophages, and classical/non-classical monocytes. (C) Stacked bar plot illustrating the proportion of neutrophil sub-clusters in the three groups (n = 3), categorized by marker genes (Ifitm6, Cers6, Ccl6, Ccrl2, Retnlg, Ltf, and Elane). (D-E) Heatmaps displaying the expression levels of signature genes for splenic immune cell clusters (D), monocyte phagocyte sub-clusters (E), and neutrophil sub-clusters (F) across WT, fas2Δ/Δ, and mock samples. The color intensity represents the expression level, highlighting differences in gene expression among specific clusters and sub-clusters between the groups. (TIF) [file ppat.1012865.s004.tif]

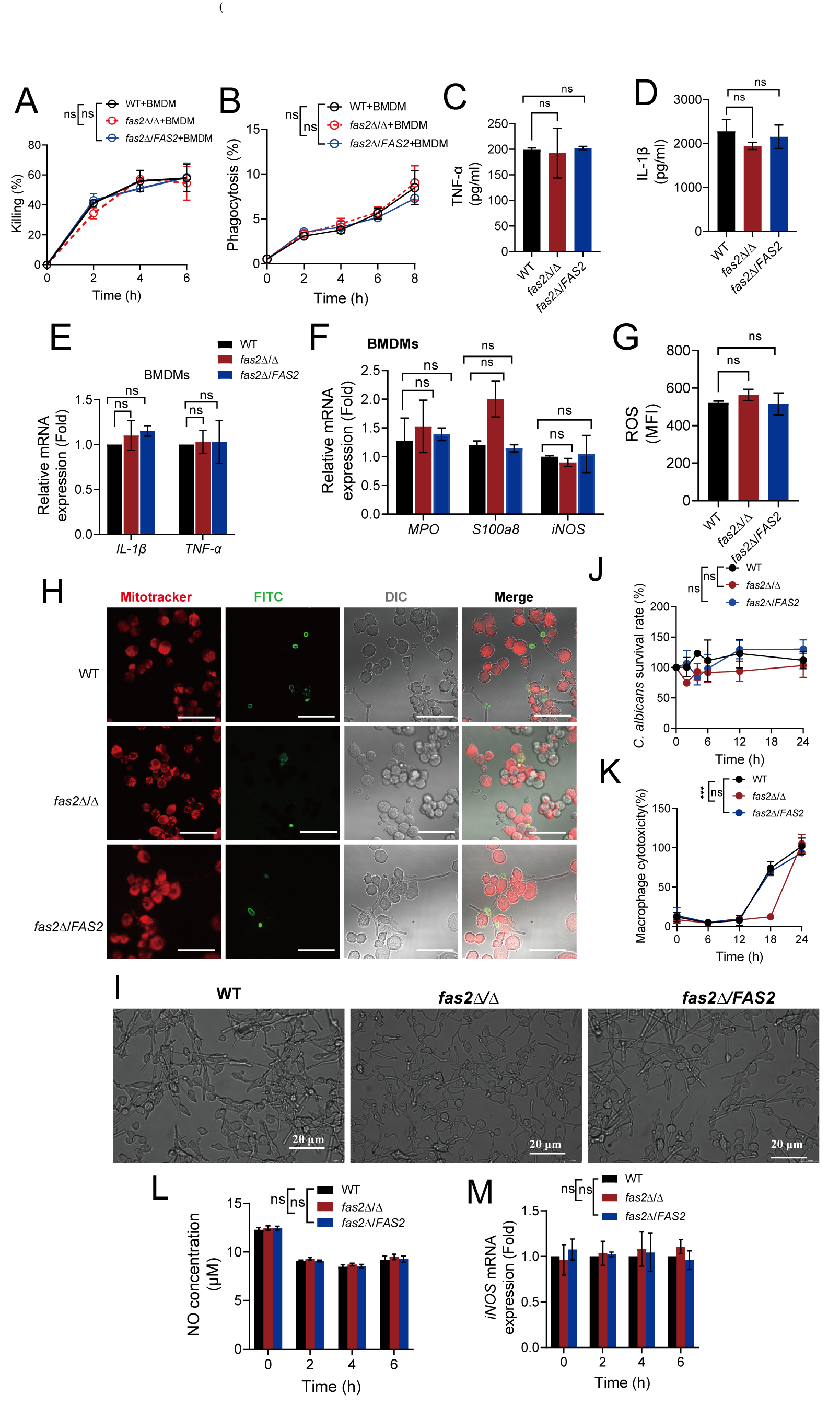

Supplement: S5 Fig — (A, B) Phagocytosis and killing capacity of BMDMs after infecting WT, fas2Δ/Δ and fas2Δ/FAS2 at a MOI=1 (n = 3). (C-E) Bone marrow-derived macrophages (BMDMs) from BALB/c mice were cultured with WT, fas2Δ/Δ and fas2Δ/FAS2 at a MOI=1 for 2 h followed by measurement of the proinflammatory cytokines levels of TNF-α (C) and IL-1β (D) in cell supernatants by ELISA (n=3), and gene expression assessment by RT-qPCR (n = 3) (E). (F) BMDMs were co-cultured with WT, fas2Δ/Δ and fas2Δ/FAS2, followed by RT-qPCR analysis of MPO, S100a8, and iNOS transcript levels (n = 3). (G) BMDMs were stimulated WT, fas2Δ/Δ, and fas2Δ/FAS2 for 2 h, followed by measurement of ROS production via a fluorescent ROS probe and the MFI analysis (n = 3). (H) RAW 264.7 macrophages preloaded with MitoTracker Deep Red FM (red) were co-cultured with FITC-stained WT, fas2Δ/Δ and fas2Δ/FAS2 cells (green). Magnification ×120. (I) The images of pretreated RAW264.7 macrophages and WT, fas2Δ/Δ or fas2Δ/FAS2 strains after 12 h co-culture at a MOI of 1:1 ratio were captured by the Leica DMi8 microscope. These images represent one of three separate experiments. (J) Survival rates of WT, fas2Δ/Δ and fas2Δ/FAS2 cells by the co-cultured with RAW264.7 macrophages. Macrophages within 24 h were determined by the endpoint dilution assay at each time point. (K) Macrophage cytotoxicity caused by WT, fas2Δ/Δ and fas2Δ/FAS2 cells were determined by the release of LDH. (L, M) Following the co-culture of RAW264.7 cells with WT, fas2Δ/Δ, and fas2Δ/FAS2 strains at a MOI of 1:1, the levels of intracellular nitric oxide (NO) and the expression of iNOS were measured. Significance is indicated as *P < 0.05, **P < 0.01, ***P < 0.001, with ns denoting no significant difference. (TIF) [file ppat.1012865.s005.tif]

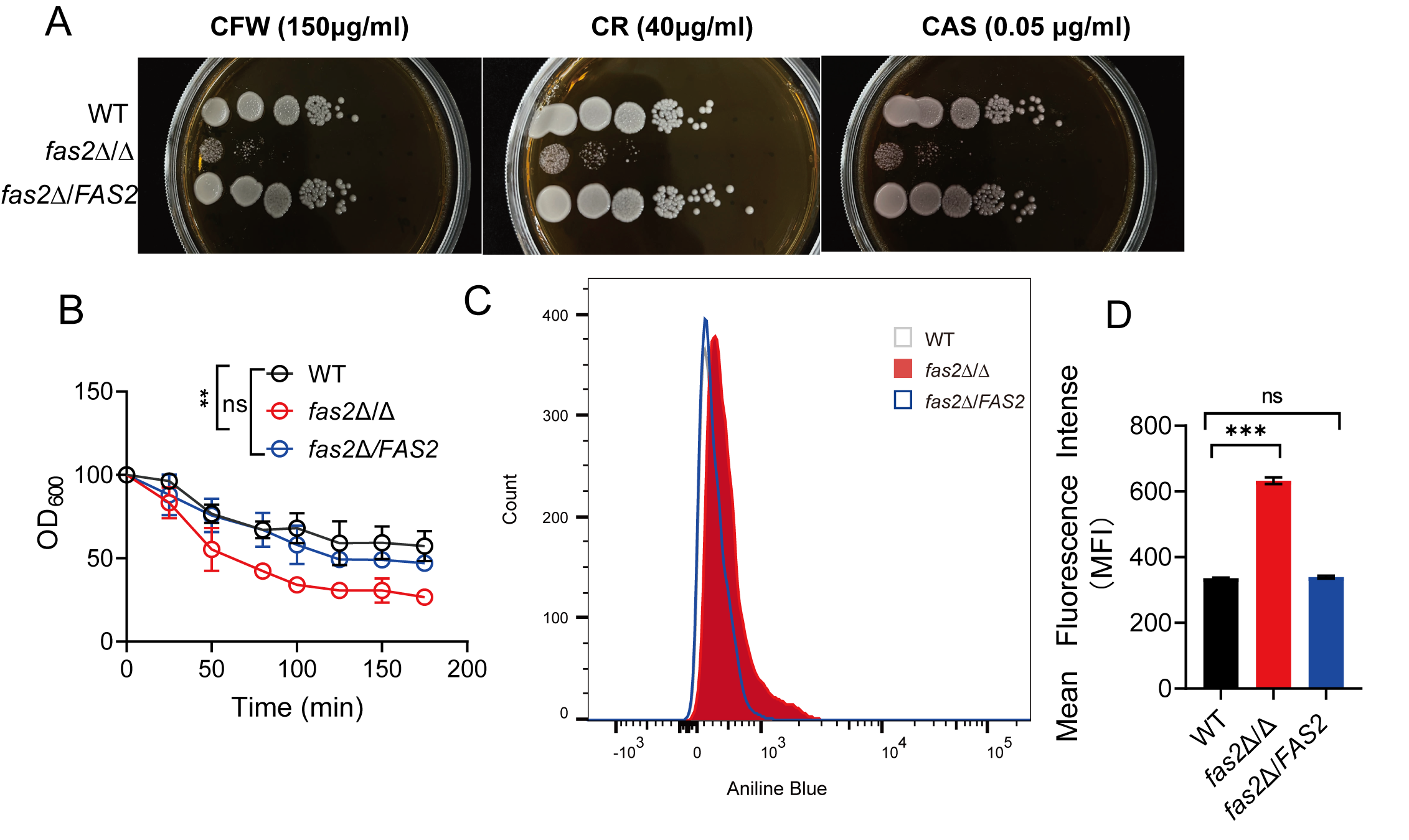

Supplement: S6 Fig — (A) Growth of WT, fas2Δ/Δ and fas2Δ/FAS2 exposed to cell wall-perturbing agents. Serial 10-fold dilutions of the cells were spotted onto YPD plates in the presence of the following agents: congo red (CR), calcofluor (CFW) and caspofungin (CAS). The plates were incubated for 48 h at 30 °C. (B) WT, fas2Δ/Δ and fas2Δ/FAS2 strains were grown to mid-log phase and incubated with recombinant β(1,3)-glucanase. The decrease in OD600 represents cell lysis as the β (1,3)-glucanase digests the cell wall and is expressed as a percentage of the starting OD600. (C, D) Quantification of Aniline Blue by FACS analysis counting 10,000 events per repeat (C). The MFI from three independent experiments is displayed with standard deviation (D). Statistical significance is denoted as **P < 0.01 and ***P < 0.001, with ‘ns’ indicating no significance. (TIF) [file ppat.1012865.s006.tif]

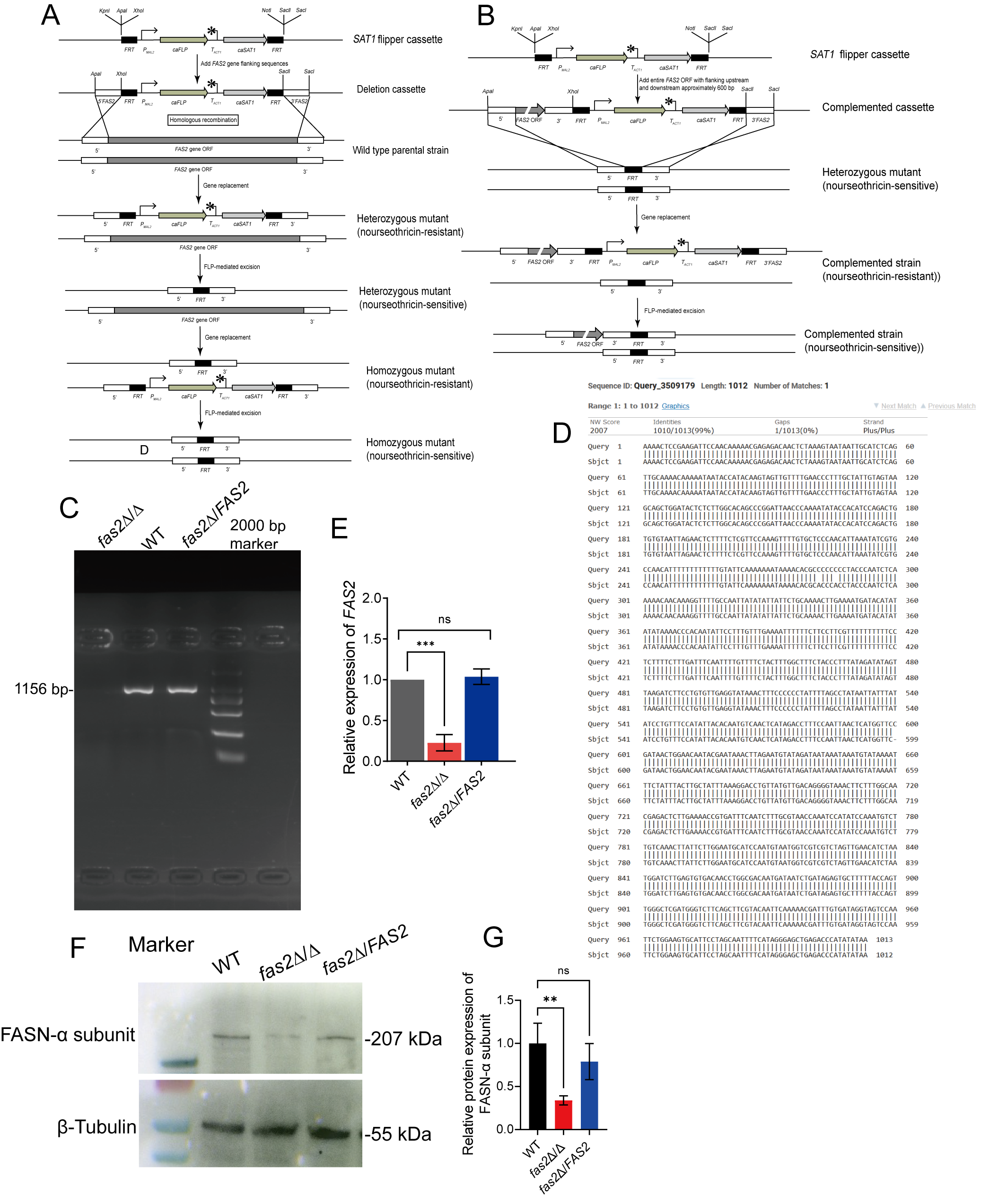

Supplement: S7 Fig — (A-D) The FAS2 gene was PCR-amplified from the genomic DNA of the WT, fas2Δ/Δ and fas2Δ/FAS2 with primers FAS2-F+FAS2-R. WT was an amplified stripe at 1156 bp; fas2Δ/Δ was without an amplified stripe; fas2Δ/FAS2 amplified stripe at 1156 bp. (E) RT–qPCR analysis of the mRNA level in FAS2 from the three strains was performed. (F) The FASN-α subunit level in WT, fas2Δ/Δ and fas2Δ/FAS2 was assessed by western blot using an anti-FAS1 antibody (provided by Guangzhou Lide Biotech). (G) Quantification of FASN-α subunit to the β-Tubulin fraction in WT, fas2Δ/Δ and fas2Δ/FAS2 strains, represented as Fold-change (n = 3 biological replicates across 3 separate blots). In E, and G, data are expressed as the mean ± SD of three independent experiments. Statistical significance is indicated by **P < 0.01 and ***P < 0.001, with ‘ns’ denoting no significance, as determined by the two-tailed unpaired Student’s t-test. (TIF) [file ppat.1012865.s007.tif]
