# Supplementary material for: Systemic infection by Candida albicans requires FASN-α subunit induced cell wall remodeling to perturb immune response
Source: PLoS Pathog. 2025 Mar 26;21(3):e1012865. doi: 10.1371/journal.ppat.1012865 (PMC11940687; doi:10.1371/journal.ppat.1012865)
Supplement: Attachment — Submitted filename: appeal letter_.docx [file ppat.1012865.s020.docx]

**Appeal Letter**

**Subject**: Appeal for Manuscript PPATHOGENS-D-24-01966

**Title**: Systemic infection by *Candida albicans* requires FASN-α subunit induced cell wall remodeling to perturb immune response

**Journal**: *PLOS Pathogens*

Dear Dr. Olszewski and Editorial Board,

We appreciate the opportunity to address the concerns raised regarding our manuscript and thank you for the detailed feedback provided by the editorial team. We have carefully revised our manuscript. Below, we provide a point-by-point response to each concern and outline the revisions made in the revised manuscript.

**1. Previous findings on *FAS2* and *C. albicans* virulence**

**Comment**: The editor noted that previous studies have already shown *FAS2*'s role in *C. albicans* virulence and that protection from subsequent infection has been observed with attenuated *C. albicans* strains.

**Response**: We acknowledge the foundational work demonstrating *FAS2*'s role in virulence. However, the precise pathogenic mechanisms remain undetermined. In fungi, studies have implicated the essentiality of *FAS2* for infectivity. Coupled with the fact that mammalian and fungal synthases are structurally distinct, this suggests that FASN-α subunit might be an appropriate target for the development of new antifungals. Based on our data, however, the activity of cerulenin, which was originally developed as an antifungal antibiotic targeting the FASN-α subunit [31954918], was completely lost against *C. albicans* infection *in vitro* with the addition of human serum. This suggests compensatory mechanisms involving exogenous fatty acids, which is consistent with the result that *fas2*Δ/Δ grew similarly to the WT strain in serum. In our revised manuscript, we have emphasized how our study builds on these findings by focusing specifically on the immunomodulatory effects of *FAS2* deletion, which leads to enhanced neutrophil-mediated immune responses through unmasking of β-glucan. We believe this focus on immune modulation rather than solely virulence provides a novel perspective that complements existing research.

**Revisions**: We have updated the Introduction and Discussion sections to clarify our unique approach and how *FAS2* advances the current understanding of *C. albicans* immunity and potential immunotherapeutic targets.

**2. Feasibility of an attenuated *C. albicans* vaccine for clinical use**

**Comment**: There was concern regarding the clinical applicability of an attenuated live *C. albicans* vaccine due to the similarity between *C. albicans* proteins and human proteins.

**Response**: We fully understand the concerns regarding the approval of live fungal vaccines that the route of priming used in our studies, as intravenous vaccine delivery is impractical for human vaccination. As the FASN-α subunit is a structurally conserved molecule in the fungal kingdom but absent in mammals [18772430], our study now aims to explore *C. albicans* *fas2*Δ/Δ might be a tool to enhance immune response and investigate trained immunity, rather than as a direct clinical vaccine candidate. In our revised manuscript, we have discussed the potential for using attenuated strains as research tools for understanding immune mechanisms and how they might inspire future immunotherapeutic strategies.

**Revisions**: We added a paragraph in the Discussion to clarify the intent of using *fas2*Δ/Δ in this research context and to propose future directions for translating these findings into safer immunotherapeutic applications.

**3. Sex of mice used in experiments**

**Comment**: The editor pointed out that the sex of the mice was not specified and suggested testing in both male and female mice.

**Response**: Thanks for your thoughtful concerns. In our revised manuscript, we have now specified the sex of the mice used (female BALB/c, 6-8 weeks old), which are commonly used experimental mice to study pathogenicity by *C. albicans* [20543849].

Furthermore, we totally agree you that the importance of including both sexes to observe potential sex-specific immune responses, because the results for males may differ. To better address whether the protective effect would also persist in male mice, our group is now trying to complete this part, and we can publish the follow up results in the future.

**Revisions**: We specified the sex of the mice in the Methods section and acknowledged the limitation and need for future studies involving both sexes in the Discussion.

**4. Potential impact of *FAS2* deletion on hyphal development**

**Comment**: The editor suggested that *fas2*Δ/Δ's avirulence might partially result from defective hyphal development and requested clarification on how *FAS2* and unmasked β-glucan influence hyphal growth.

**Response**: Thanks for your insightful comments. In *C. albicans*-macrophage co-culture assays, we observed that the majority of *fas2*Δ/Δ either formed small hypha-like outgrowths or failed to undergo detectable growth within the macrophag by 2 h (**Fig. S5H**); however, after 12 h, the mutant formed hyphae and escaped from macrophages, resulting in increased *C. albicans* survival rates and macrophage cell damage (**Fig. S5I-K**). Furthermore, our data confirm that *fas2*Δ/Δ displays normal hyphal formation under serum-supplemented conditions. The result of *fas2*Δ/Δ is dispensable for morphogenesis under *in vitro* conditions but not in the host, suggests that the avirulence were not substantially associated with major virulence factors of *C. albicans.* To address this more thoroughly, we have expanded the discussion of *FAS2*'s role in hyphal development and how unmasking β-glucan enhances immune detection without directly impairing hyphal morphology.

**Revisions**: We have rewritten this section to explain the relationship between hyphal development and the avirulence of *fas2*Δ/Δ *in vivo*, and to discuss the implications of β-glucan exposure in immune responses rather than morphogenesis.

**5. Supplementation with key fatty acids to rescue *fas2*Δ/Δ defects**

**Comment**: The editor recommended supplementation with key fatty acids to assess if this could rescue the effects of *FAS2* deletion.

**Response**: We appreciate this insightful suggestion and have included relevant findings in the revised manuscript. According to the published papers, the *fas2*Δ/Δ strain in our study was propagated in fatty acid-containing medium that included YPD plus 0.01% (w/v) of myristic acid (14:0), palmitic acid (16:0), and 1% (v/v) of Tween 40 [9009352] with shaking at 30 °C overnight. The predominant fatty acids in human serum, comprising ~80% of the total fatty acids, are the unsaturated linoleic (C18:2n-6) and oleic (C18:1n-9) acids, and the saturated palmitic (C16:0) and stearic acids (C18:0) [12575911]. Although we did not conduct additional experiments with individual fatty acids composed in human serum, we showed that the addition of serum restored the fatty acid composition of *fas2*Δ/Δ to levels more like WT using liquid chromatography/mass spectrometry (LC/MS) (**Fig. 5F-I**), indicating that exogenous fatty acids in human serum can compensate for the fitness defects of *fas2*Δ/Δ.

**Revisions**: We have included the serum supplementation results in the main text, along with references supporting the fatty acid composition of serum and its relevance to *C. albicans* growth.

**6. Missing Figure 7**

**Comment**: The editor noted that Figure 7 appeared to be missing, which was intended to demonstrate the protective effect of the *fas2*Δ/Δ strain.

**Response**: Correction has been made in our revised manuscript. Figure 7 has now been included in the manuscript, clearly displaying the protective effect of *fas2*Δ/Δ against subsequent virulent *C. albicans* infection. This figure strengthens our conclusions regarding the trained immunity effect elicited by *fas2*Δ/Δ.

**Revisions**: Figure 7 has been added, along with a description in the Results section detailing the experimental setup and findings related to protective immunity.

**7. Additional clarity and minor revisions**

Throughout the manuscript, we have made minor revisions to improve clarity and readability. We have also ensured that all figures and supplementary data are properly labeled and cross-referenced within the text.

Thank you again for considering our response and revised manuscript. We believe these revisions address all the concerns raised and strengthen the manuscript. We hope the revised version will meet the standards of *PLOS Pathogens* and contribute valuable insights to the field of fungal immunotherapy.

Please feel free to reach out if you require further clarification on any of our responses or revisions.

Thank you for your time and consideration.

Sincerely,

Yajing Zhao

~~~~~~~~~~~~~~~~~~~~

Department of Dermatology,

The First Affiliated Hospital of Jinan University,

Guangzhou 510630, P.R. China

[Tel: +86-020-852-23180](Tel:+86-020-852-23180)

E-mail: [zhaoyj@jnu.edu.cn](mailto:zhaoyj@jnu.edu.cn;)
