# Supplementary material for: Systemic infection by Candida albicans requires FASN-α subunit induced cell wall remodeling to perturb immune response
Source: PLoS Pathog. 2025 Mar 26;21(3):e1012865. doi: 10.1371/journal.ppat.1012865 (PMC11940687; doi:10.1371/journal.ppat.1012865)
Supplement: Attachment — Submitted filename: Response to Reviewers.docx [file ppat.1012865.s021.docx]

**Cover letter**

Dear editors and reviewers,

Attached please find the revised version of our manuscript entitled “Systemic infection by *Candida albicans* requires FASN-α subunit induced cell wall remodeling to perturb immune response” submitted to your journal of “***PLOS Pathogens***” (PPATHOGENS-D-24-01966). We appreciate the reviewers’ comments and insightful criticism, which significantly improved the quality of our work. We have extensively revised our manuscript following their comments and suggestions. All the changes are marked in red and point-by-point response below are in blue. Thank you for your kindly consideration on our work. Please contact us if any further information is needed.

Best regards,

Yours Sincerely,

Yajing Zhao

Jinan University

#601 West Huangpu Ave, Guangzhou, Guangdong, China. 510632

Email: [zhaoyj@jnu.edu.cn](mailto:zhaoyj@jnu.edu.cn;)

**Comments and Responses**

Part I - Summary
Reviewer #1: (No Response)

Reviewer #2: The manuscript by Zhao et al presents data on the role of fatty acid synthase gene FAS2 in Candida albicans virulence. The FAS2 gene has previously been shown to be essential for systemic as well as oropharyngeal candidiasis. In the present study the authors have attempted to uncover the mechanisms behind avirulent phenotype of *fas2*Δ/Δ null mutant. The authors show that the survival of *fas2*Δ/Δ mutant strain in immunocompetent mice was abolished in cyclophosphamide treated mice. From a series of experiments the authors show that neutrophils are the primary cell type that mediate *C. albicans* *fas2*Δ/Δ killing. The role of neutrophils in increased *fas2*Δ/Δ killing was further supported by increased b-glucans exposure. Finally, the authors performed experiments to show the vaccine potential of FAS2. Overall, the findings of this work are interesting, and may help better understand the contributions of FAS2 in fungal pathogenesis. The manuscript is well written. The methodology is sound and in most cases the results justify the conclusions.

*Our response*:

We are grateful for the positive feedback from reviewer .

Part II – Major Issues: Key Experiments Required for Acceptance
Reviewer #1: The group did not address the concern from prior review about the use of female mice only.

Reviewer #2: (No Response)

*Our response*:

We appreciate the reviewers’ concerns regarding the exclusive use of female mice in our previous studies. To address these concerns, we conducted the following experiments with male mice. The 8 male BALB/c mice (6-8 weeks old) in each group were intravenously infected with either 5 × 10^5^ CFU of WT, *fas2*Δ/Δ, *fas2*Δ/*FAS2*, or 5 × 10^6^ CFU of *fas2*Δ/Δ, with 100 µl saline injection as a control. As shown in **Fig 1**, the survival rate of the male mice after 15 days was 100%. Furthermore, etiologic analysis revealed that the fungal burdens in kidneys, livers and spleens of male mice infected with *fas2*Δ/Δ were significantly decreased at 48 hours post-infection. Histopathologic examination of the kidneys confirmed that the male mice infected with *fas2*Δ/Δ displayed normal tissue morphology, with no evidence of tissue damage, hyphae, pseudohyphae, spores, or inflammatory cell infiltration. These findings were congruent with the results observed in female mice. Collectively, these findings robustly indicate that *fas2*Δ/Δ elicits a comparable immune response in both male and female mice, highlighting the conservation of immune responses across genders in the context of *fas2*Δ/Δ virulence.

**Figure 1. The *fas2*Δ/Δ mutant is avirulent in male mice.** (A) Survival curves of male BALB/c mice (6-8 weeks old) (n=8) after intravenous injection with *C. albicans* WT, *fas2*Δ/Δ, *fas2*Δ/*FAS2* at 5×10^5^ CFUs per mice, and *fas2*Δ/Δ at 5×10^6^ CFUs per mice. (B) *C. albicans* fungal burdens (CFU per g of tissue) in organs at 48 h postinfection with WT, *fas2*Δ/Δ and *fas2*Δ/*FAS2* (5 × 10^5^ CFUs per mice) (n=3)*.* (C) Representative images of PAS stained kidney sections of mice 72 h after systemic infection with WT, *fas2*Δ/Δ and *fas2*Δ/*FAS2*. Scale bars, 50 µm. Data in (A, and B) are presented as mean ± SD. Statistical significance was determined using the log-rank test for survival curves (A), and two-tailed unpaired Student’s t-test for fungal burdens (B). Significance is indicated as **P*<0.05, ***P*<0.01, ****P*<0.001, with ns denoting no significant difference.

Part III – Minor Issues: Editorial and Data Presentation Modifications

Reviewer #1: The group discusses the use of live *Candida* as a vaccine in the discussion. Prior review recommended that this be edited as the feasibility for a live Candida vaccine is low.

*Our response*:

We thank the reviewers for their insightful comments. Following the reviewers’ recommendations, we have decided to remove the relevant information pertaining to the live *Candida* vaccine from the ‘Discussion’ section.

Reviewer #2: 1. Figure 6C, In the Mkc1 and Cek1 phosphorylation western blots the mkc1Δ/Δ and cek1Δ/Δ null mutant control strains are missing. The tubulin loading control also needs to be repeated.
2. Figure 6E, the quality of Rho1 western blot is very poor.

*Our response*:

1) We sincerely appreciate the reviewer’s comments and suggestions. We agree that incorporating *mkc1*Δ/Δ and *cek1*Δ/Δ mutants as controls would strengthen the evidence for the activation of the Mkc1-MAPK cascade following *FAS2* disruption. However, Since the time we conducted western blot assay, *mkc1Δ/Δ* and *cek1Δ/Δ* mutants have not been available in our laboratory. Generation of these mutants would require significant time and resources, which could lead to a significant delay in the dissemination of our findings.
2) Our current experiments are preliminarily focused on evaluation of the effects of *fas2*Δ/Δ on the activation (phosphorylation) of Mkc1 and Cek1 in *C. albicans*, specifically in relation to cell wall phenotypes that have been demonstrated by many studies (References below: Wagner AS, et al and Chen, et al). Considering the constrains associated with mkc1Δ/Δ and cek1Δ/Δ mutants, we are unable to conduct the additional experiments suggested at this time. We apologize for any shortcomings in addressing this matter and are committed to testing this concern once the mutants are available. Meanwhile, we have revised the manuscript to update the corresponding tubulin loading control image in Fig. 6C. We have included a representative image selected from three independent Western blot experiments to ensure accuracy and consistency.

Nevertheless, we greatly appreciate the review’s valuable insights and are dedicated to further exploring this aspect in forthcoming research endeavors.

3) We thank the reviewer for pointing this out. Following the established method by Borah et al., who employed the Rho Activation Biochem Kit (Cytoskeleton, BK036) to assess Rho1 activation in *Candida glabrata* (Borah et al., 2011). We have adopted the same kit to demonstrate an increase in GTP-Rho1 activity in *fas2*Δ/Δ compared to that of WT in our experiments. However, we recognize the suboptimal quality of the Rho1 western blot. We reasoned that this affinity purification assay offers a straightforward approach to analyze cellular Rho activities across various systems, which has been recommended to be broadly applicable across species. This implies that while the kit is versatile, its specificity for use in *C. albicans* might be compromised. We have taken this feedback into account and will explore alternative or supplementary methods in future studies to enhance the quality of data presentation regarding Rho1 activation in *C. albicans*.

**References in this part:**

Wagner AS, Hancock TJ, Lumsdaine SW, Kauffman SJ, Mangrum MM, Phillips EK, et al. Activation of Cph1 causes ß(1,3)-glucan unmasking in *Candida albicans* and attenuates virulence in mice in a neutrophil-dependent manner. PLoS Pathog. 2021;17(8):e1009839. PMID: 34432857.

Chen T, Wagner AS, Tams RN, Eyer JE, Kauffman SJ, Gann ER, et al. Lrg1 Regulates β (1,3)-Glucan Masking in *Candida albicans* through the Cek1 MAP Kinase Pathway. mBio. 2019;10(5):e01767-19. PMID: 31530671.

Borah S, Shivarathri R, Kaur R. The Rho1 GTPase-activating protein CgBem2 is required for survival of azole stress in *Candida glabrata.* J Biol Chem. 2011;286(39):34311-24. PMID: 21832071.
